# Researchers’ attitudes to the 3Rs—An upturned hierarchy?

**DOI:** 10.1371/journal.pone.0200895

**Published:** 2018-08-15

**Authors:** Nuno Henrique Franco, Peter Sandøe, I. Anna S. Olsson

**Affiliations:** 1 i3S - Instituto de Investigação e Inovação em Saúde, Universidade do Porto, Porto, Portugal; 2 IBMC – Instituto de Biologia Molecular e Celular, Universidade do Porto, Porto, Portugal; 3 Department of Veterinary and Animal Sciences, University of Copenhagen, Copenhagen, Denmark; 4 Department of Food and Resource Economics, University of Copenhagen, Copenhagen, Denmark; Public Library of Science, UNITED KINGDOM

## Abstract

Animal use in biomedical research is generally justified by its potential benefits to the health of humans, or other animals, or the environment. However, ethical acceptability also requires scientists to limit harm to animals in their research. Training in laboratory animal science (LAS) helps scientists to do this by promoting best practice and the 3Rs. This study evaluated scientists’ awareness and application of the 3Rs, and their approach to other ethical issues in animal research. It was based on an online survey of participants in LAS courses held in eight venues in four European countries: Portugal (Porto, Braga), Germany (Munich, Heidelberg), Switzerland (Basel, Lausanne, Zurich), and Denmark (Copenhagen). The survey questions were designed to assess general attitudes to animal use in biomedical research, Replacement alternatives, Reduction and Refinement conflicts, and harm-benefit analysis. The survey was conducted twice: immediately before the course (‘BC’, N = 310) and as a follow-up six months after the course (‘AC’, N = 127). While courses do appear to raise awareness of the 3Rs, they had no measurable effect on the existing low level of belief that animal experimentation can be fully replaced by non-animal methods. Most researchers acknowledged ethical issues with their work and reported that they discussed these with their peers. The level of an animal’s welfare, and especially the prevention of pain, was regarded as the most pressing ethical issue, and as more important than the number of animals used or the use of animals as such. Refinement was considered more feasible than Replacement, as well as more urgent, and was also favoured over Reduction. Respondents in the survey reversed the ‘hierarchy’ of the 3Rs proposed by their architects, Russell and Burch, prioritizing Refinement over Reduction, and Reduction over Replacement. This ordering may conflict with the expectations of the public and regulators.

## 1. Introduction

Animal experimentation is controversial. However, despite the strong opposition of a small but vocal segment of society, most people in countries where attitudes to it have been studied have been found to regard it as legitimate as long as two conditions are met: first, it must have real potential to deliver findings that enable us to address problems involving human health, animal health, or the environment; and second, animal welfare needs must be looked after in the best way possible [1,2,3]. This widely shared view is reflected in most of the legislation currently regulating animal use.

The ‘social licence’ scientists have to perform animal experiments requires researchers to do more than merely comply with laws and regulations [4], because while public attitudes help to shape the legal and regulatory framework in which scientists work, in practice the framework gives considerable freedom to scientists. By and large, the public trust scientists to exercise this freedom in acceptable ways. However, relatively little is known about whether the values and attitudes of scientists working with animals align with those of the society of which they are a part.

The most widely accepted guidance on limiting harms to animals in biomedical research is given by the principles of the 3Rs proposed by William Russell and Rex Burch in the late 1950s: Replacement, Reduction and Refinement [5]. Today these principles are embedded in many laws, regulations and codes governing laboratory animal use [6]. In essence, they allow animals to be used in scientific research only when they cannot be *replaced* with non-animal alternatives, when the number of animals has been *reduced* as much as possible given the research goals, and when procedures and housing have been *refined* to minimize welfare impacts.

The 3Rs often improve the quality of animal studies. Reduction can lead to the use of more sophisticated project design and analysis, for example, and Refinement might minimize the confounding effect of comorbidities arising from animal health and welfare problems secondary to the modelled disease [7,8,9,10,11,12]. Such incidental effects are important, because as well as being ethically acceptable, animal research must actually deliver its promised benefits. However, scientific validity and concerns about 3Rs may also conflict in several ways. For example, the principle of Reduction requires animal numbers to be minimized, but published meta-analyses have shown that many studies are in fact underpowered and do not use a sufficient number of animals for the results to be reliable [13]. Tensions can also arise between the 3Rs. In some circumstances experimental refinements minimizing animal suffering are only feasible if a larger number of animals are used [11,14,15].

To obtain a formal license to carry out research with animals, scientists must undergo appropriate training. Education and training are needed to raise awareness of animal welfare issues and to improve levels of competence in implementation of the 3Rs [16,17,18,19,20]. Education here involves ‘the didactic presentation of the information and theories of animal use that will contribute to the development of proper attitudes toward the use of animals’. Training is the ‘acquisition of practical knowledge and skills directly associated with animal handling and procedures’ [20].

Mandatory courses in laboratory animal science (LAS) aim to promote competence and raise standards of animal welfare [21,22]. The standards most widely used in LAS education and training were set by the FELASA (Federation for Laboratory Animal Science Associations) recommendations for categories B[23] and C[24]. These recommendations are now being absorbed and adapted [25] within the EU ‘functions system’ introduced by the 2010/63/EU Directive [26] on the protection of animals used for scientific purposes. The recommendations explicitly state that the 3Rs must be included in training curricula. However, the training of life science researchers has also been criticized for reinforcing and legitimizing a belief in the inescapability of animal experiments. It is alleged that, intentionally or unintentionally, this message is part of a ‘hidden curriculum’ in LAS courses [27,28].

With this background in mind, we surveyed a broad and diverse sample of researchers undergoing training in laboratory animal science in four European countries. Our aim was to answer the following questions:

- How do scientists perceive the need for animal use and its ethical justification?
- How aware of the 3Rs are scientists working, or intending to work, with animals?
- What attitudes to the 3Rs do animal scientists have?
- How do animal scientists rate the relevance and utility of what is taught in LAS courses?

## 2. Materials and methods

The development of the survey started with writing a guiding document outlining the background, which included results from a previous survey [29], and established the rationale for the current study. Based on this, research questions were proposed, and several hypotheses stemming from the previous survey were enunciated. Questionnaire items were then developed to test each of the aforementioned hypotheses. After the guiding document and a first version of the survey had been thoroughly discussed between the authors, a pilot study was carried out to test-run it, in December 2013, in Braga. Respondents in the pilot study answered the questionnaire online and were asked during the LAS course (where two of the authors, NF and AO, gave classes) to give their feedback on it, regarding easiness of use, readability, clarity, length and difficulty. Following this, the questionnaire was refined using the information gathered and feedback from the volunteer participants. As the final step, input was sought from a social sciences researcher on the guiding document and the revised version of the questionnaire. We then proceeded with recruiting collaborators from across Europe.

We contacted LAS course organizers across Europe to collaborate in our survey. We approached colleagues with whom we had contacts, and also called for collaborators at a workshop on LAS education held in Varese, Italy, 30–31 October 2014. We received replies from organizers running FELASA B (40-hour courses for people carrying out scientific procedures in animals) and FELASA C (80-hour courses covering more ground than FELASA B courses which are recommended for researchers intending to design, carry out or supervise animal studies). Those responding to our call were employed in four European countries: Portugal, Switzerland, Denmark and Germany.

Overall, 14 courses in venues in eight cities were surveyed: Braga (one C course), Porto (three C courses), Munich (one B and one C course), Heidelberg (one B course), Copenhagen (one C course), Zurich (four B courses), Basel (one B course) and Lausanne (one B course). All of the courses were held between May 2014 and July 2015. The course organizers agreed to forward an online questionnaire in English (see supplementary material) to their course participants, and to do this again six months later in a follow-up survey. Oversight by internal ethics committees was nowhere required, since participation was fully anonymous and voluntary. Students were assured during the recruitment and before starting the questionnaire that neither participation nor their answers would have any impact on their course evaluation.

The questionnaires sent out six months after the course (AC) were identical to those used in the survey carried out before the course (BC). However, the AC questionnaires were sent together with a set of additional questions asking respondents to self-evaluate their experience of the courses (S1 Questionnaire Example). The questionnaire was divided in two parts. The first investigated respondents’ level of awareness of the 3Rs without introducing or elaborating the principles. The second part opened with a description of the principles to allow those with no understanding of the 3Rs to reply to questions about them. Some participants (27% BC and 22.9% AC) replied to the first part of the questionnaire but not the second. We therefore provide data on sample size for questions answered in the second part.

**Fig 1:**
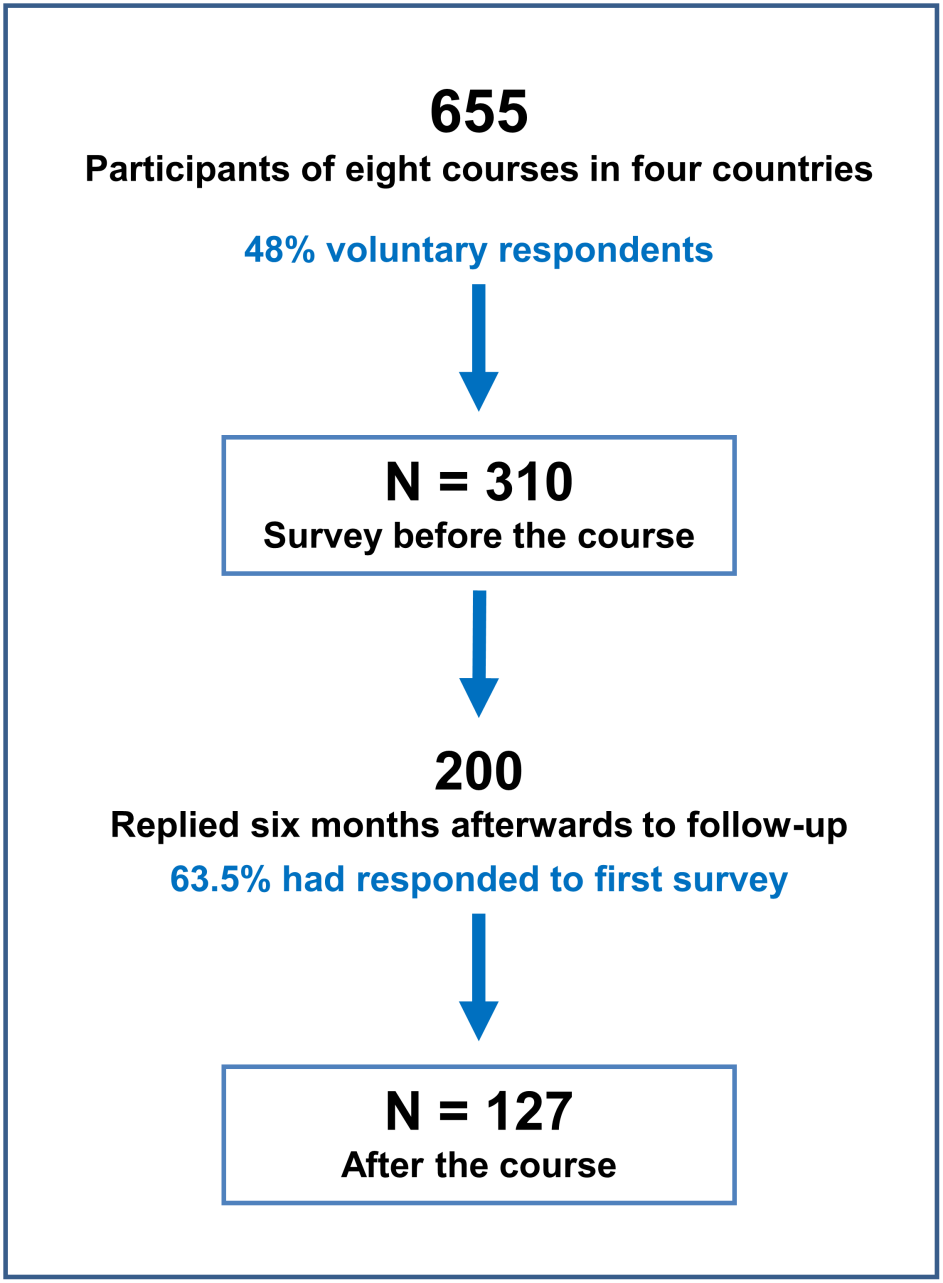
Sampling and attrition rate.

Of the 655 course participants who were asked to respond, 310 fully, or partly, completed the first-round survey run at the start of the course. Although 200 took part in the follow-up six months later, only the 127 who confirmed that they had participated in the first-round survey were included in the analysis (Fig 1).

To analyse the potential effect of age on the other parameters evaluated, age was transformed into an ordinal variable split into five groups (20–25; 26–30; 31–35; 36–40 years; and >40 years). Each survey respondent was defined as an experimental unit. Anonymity meant that respondents from the BC and AC surveys could not be matched, so tests of statistical significance were not applied in inter-survey comparisons (groups were neither paired, on one hand, nor independent, on the other). For other factors—gender, age, country, and venue—Pearson chi-square tests were used for nominal variables and Mann-Whitney tests for ordinal variables. Differences were deemed significant for p<0.01. The statistical analysis was performed using the IBM-SPSS 23 statistical package.

## 3. Results

### 3.1. Sample characterization

Demographics for the first- and second-round surveys were similar as regards age distribution and gender composition, and proportionate as regards type of course attended, country where course was held, nationality, occupation, topic of the respondent’s first degree, topic of respondent’s PhD, and support for animal protection organizations (detailed data provided as S1 Table). Most respondents (63.2%) reported that they had had experience with laboratory animals prior to taking the survey, with the proportion being higher (82.7%) in the second-round survey six months later.

Mean age in the first-round survey was 30.8 years (SD = 5.2), ranging from 21 to 55 years. There was a similar distribution in the follow-up survey (mean age of 30.9, SD = 5.6). Age was not significantly different between countries or venues, with the exception of the Braga course (N = 14), where the mean age (23.7 years) was lower than in most other venues (and significantly so for Porto and Munich, p<0.01, one-way ANOVA, Tukey adjustment post-hoc for multiple comparisons) because laboratory animal science courses there are run as curricular modules in some M.Sc. and Ph.D. courses (see S1 Fig).

Women outnumbered men in the overall sample (Table 1), but gender composition ranged from 75% women in Braga to 48% in Copenhagen, where uniquely there were more male than female respondents.

**Table 1:** Attitudes to Replacement, Reduction and Refinement. Respondents scored their agreement with statements about Replacement alternatives on a Likert scale (‘strongly disagree’; ‘disagree’, ‘neither agree nor disagree’; ‘agree’; ‘strongly agree’). Percentages of respondents either agreeing (‘agree’ and ‘strongly agree’) or disagreeing with the statements (‘disagree’ and ‘strongly disagree’) are clustered). The option selected by most respondents (‘mode answer’) is highlighted for each statement and survey (N = 229–234 BC, N = 97–98 AC, due to varying attrition rates).

		Disagree %	Neither agree nor disagree %	Agree %	Not sure %
My research protocols have sufficient consideration for the 3Rs	BC	1.7	12.0	70.5	15.8
	AC	0.0	14.3	78.6	7.1
I intend to further implement the 3Rs in my work	BC	1.7	5.1	89.3	3.8
	AC	3.1	8.2	84.7	4.1
I do not know about the 3Rs as much as I want to.	BC	21.8	25.3	46.8	6.0
	AC	66.3	19.4	13.3	1.0
I find any animal experiment acceptable, provided the 3Rs are fully considered	BC	15.8	15.5	56.5	12.2
	AC	33.7	12.2	51.0	3.1
I have no issues with relevant and scientifically sound animal experiments, even if the 3Rs are not fully considered	BC	67.5	14.2	7.1	11.2
	AC	77.6	11.2	8.1	3.1
‘Refinement’ measures are a prerequisite for the quality of animal research	BC	2.1	3.4	88.5	6.0
	AC	1.0	3.1	94.9	1.0
‘Refinement’ measures can negatively interfere with the reproducibility of animal experiments	BC	51.7	19.2	12.0	17.1
	AC	62.2	20.4	13.3	4.1
How animals are treated is more important than how many animals are used	BC	14.2	24.5	53.7	7.7
	AC	12.2	29.6	55.1	3.1
Full Replacement of animal experimentation can be achieved in the foreseeable future	BC	67.0	10.4	9.1	13.5
	AC	76.3	13.4	7.2	3.1
There is room for some Replacement but ultimately, animal experiments will always be necessary	BC	4.3	7.0	81.7	7.0
	AC	7.2	10.3	77.3	5.2
Non-animal methods have their own place and value in biomedical research, and should not be seen as mere alternatives to animal experiments	BC	13.0	13.0	70.4	3.5
	AC	11.3	20.6	65.0	3.1
The end to animal experiments will only be possible when effective treatments are available for all known diseases	BC	37.0	25.7	20.4	16.1
	AC	35.0	29.9	22.7	12.4
The most effective step to reduce the number of animals used would be to apply more rigorous criteria regarding which projects merit approval	BC	17.5	23.1	49.3	10
	AC	21.6	23.7	52.6	2.1
Full ‘Refinement’ of animal experiments is more urgently needed than their Replacement	BC	11.4	16.6	62.4	9.6
	AC	12.4	17.5	70.1	0
Whether animal experiments can be replaced or not will depend on the level of scientific and technological development of alternative methods	BC	10.0	10.9	72.1	7.0
	AC	7.2	10.3	80.4	2.1
Full ‘Refinement’ of animal experiments is a more readily achievable goal than Full ‘Replacement’	BC	3.9	9.6	80.0	6.6
	AC	2.1	9.3	87.6	1.0
Having results from animal studies makes it easier to publish research in a high-ranking journal	BC	14.4	21.4	53.7	10.5
	AC	10.3	18.6	63.9	7.2
Using fish, rather than mammals (such as mice) is a relevant ‘Replacement’	BC	59.0	21.8	8.3	10.9
	AC	49.5	19.6	26.8	4.1
In my case, replacing animal experiments for non-animal alternatives would be too expensive	BC	34.5	40.2	4.8	20.5
	AC	45.4	27.8	7.2	19.6
Using invertebrates (other than cephalopods), rather than vertebrate animals, constitutes relevant ‘Replacement’	BC	51.5	21.0	17.9	9.6
	AC	36.0	26.8	29.9	7.2

Respondents on the Braga, Munich, and Porto courses were mainly nationals of the countries in which the course was held, with only 0%, 15%, and 17%, respectively, being foreign. On all other courses, the respondents were mostly foreign nationals: 69% in Lausanne, 91% in Basel, 71% in Zurich, 74% in Heidelberg, and 64% in Copenhagen. Overall, 55.2% of BC respondents and 56.7% of AC respondents had a nationality differing from that of the country in which the course was being held. The total sample included respondents from 42 countries (26 AC). The largest proportion were Portuguese (19.4% BC, 16.5% AC), followed by Swiss (17.1% BC, 15.0% AC), German (16.8% BC, 25.0% AC), and Italian nationals (11.0% BC, 9.4% AC), with non-negligible numbers of Danish (2.3% BC, 3.1% AC), French (3.9% BC, 4.7% AC) and Indian nationals (3.5% BC 3.9% AC). In total, the remaining nationalities (mostly European) made up 26.1% of sample BC and 26.7% of sample AC.

### 3.2. Scientific need and ethical concerns regarding animal use

**Fig 2:**
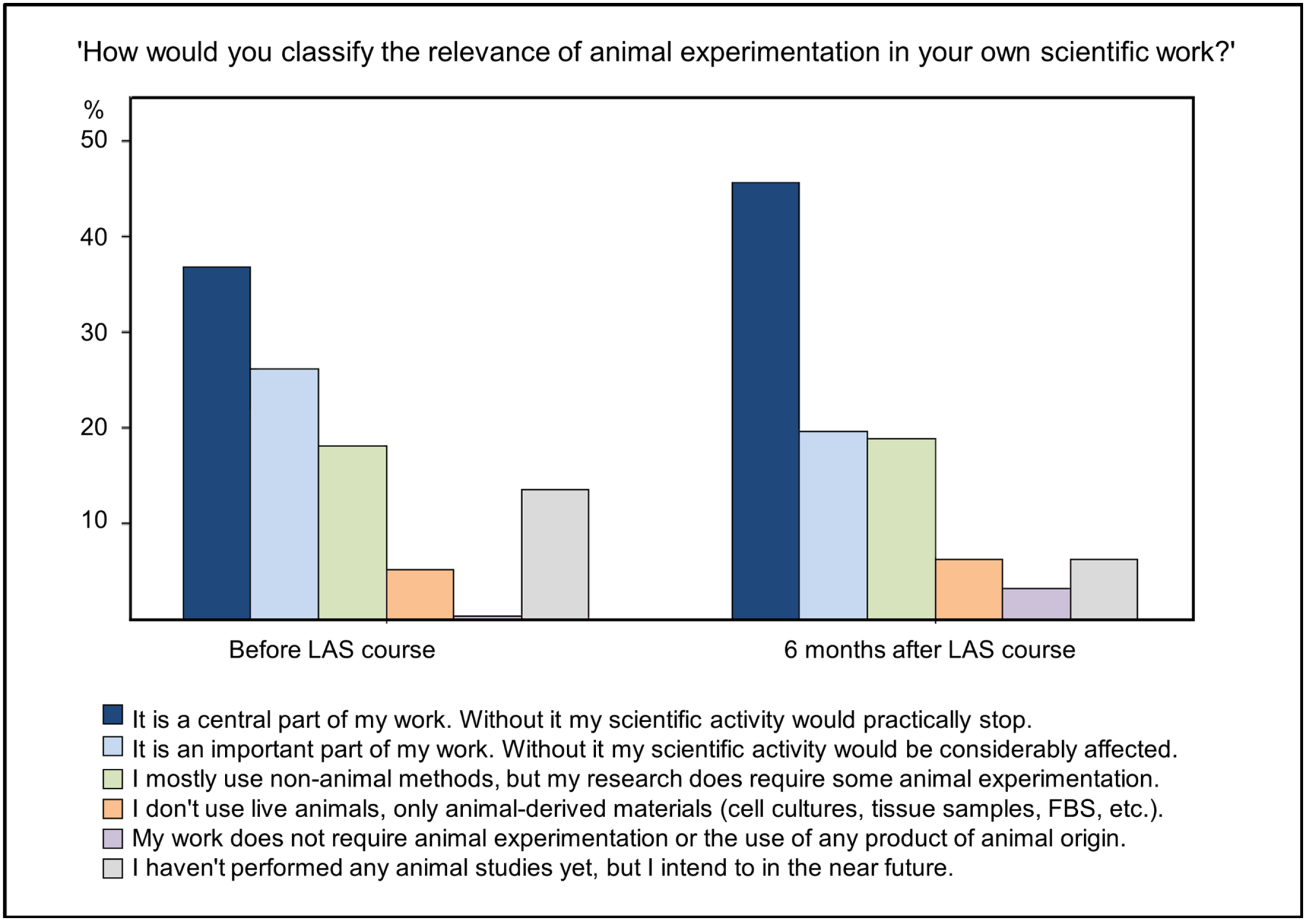
Self-assessed relevance of animal experimentation in scientists’ own work.

When asked ‘How would you classify the relevance of animal experimentation in your own scientific work?’, only 1 BC respondent and 4 AC respondents stated that their work required neither live animals nor animal-derived products (Fig 2). However, those who did not work with animals at the time of the questionnaire stated that they intended to do so in the future. In all, 196/310 (63.2%) BC and 106/127 (82.7%) AC indicated that they already had experience of working with laboratory animals. Of these, 77.6% (74.5% AC) considered it a ‘central’ or an ‘important’ part of their work, while 16.3% BC and 18.9% AC mostly used non-animal methods but were required to do some animal experimentation, and 3.1% BC and 4.7% AC used animals only indirectly through use of animal-derived materials.

Asked whether there were any research steps presently requiring animals that could potentially be replaced with a non-animal approach, 33% (21.3% AC) stated that they had not yet worked with animals. Of those remaining, 82.6% (83.0% AC) considered there were no Replacement alternatives for any steps in their experiments.

**Fig 3:**
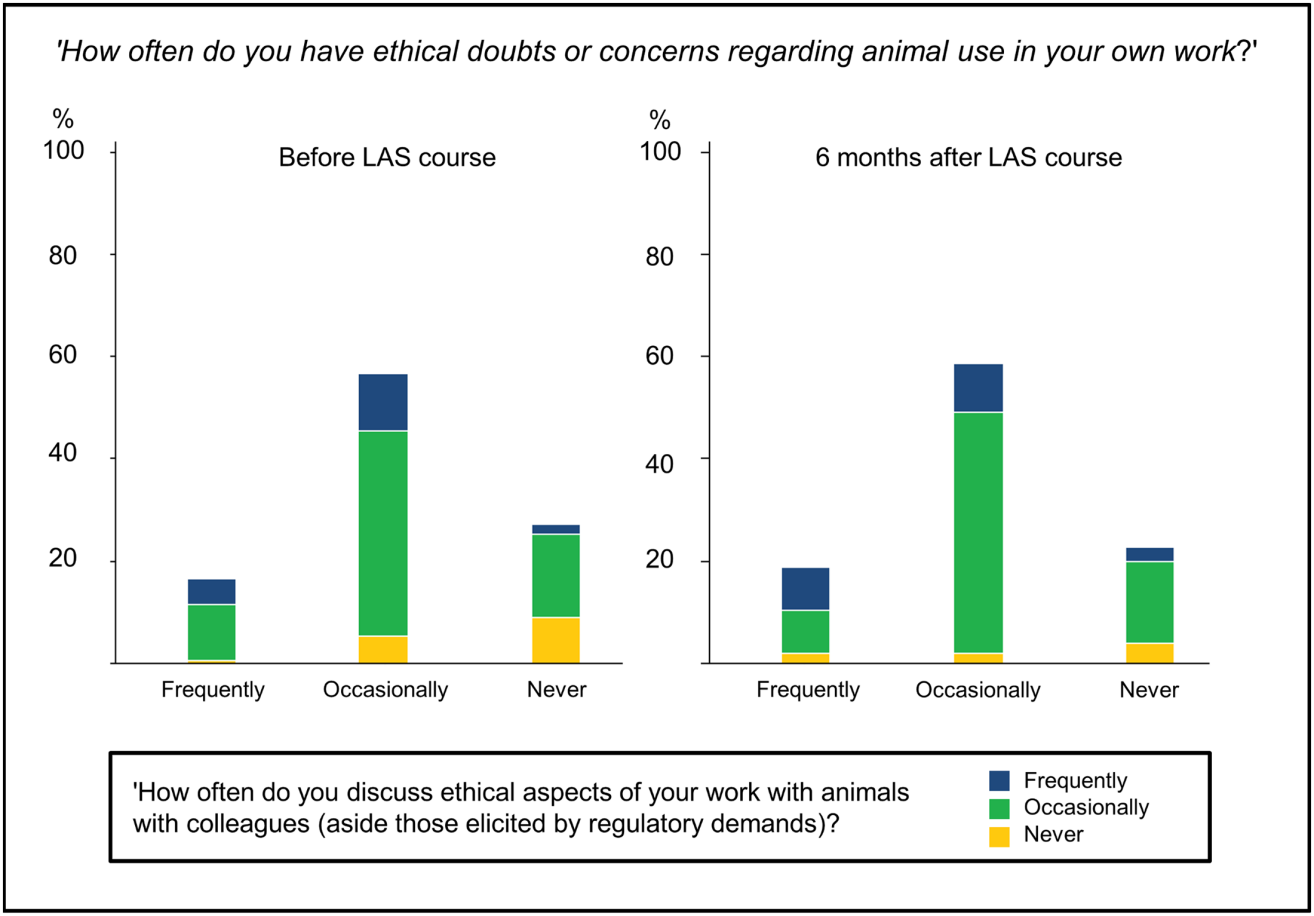
Ethical concerns about animal use in relation to self-reported frequency with which these concerns are discussed with colleagues.

In response to the question ‘How often do you have ethical concerns regarding animal use in your own work’, 28.4% stated that they had not yet worked with animals (16.5% AC). Of those working with animals, only 27.4% (22.6% AC) indicated that they had never had ethical concerns about their work, 16.1% (18.9% AC) reported having frequent ethical concerns and 56.5% (58.5% AC) reported having such concerns occasionally. Many of the respondents already working with animals confirmed that they had discussed ethical aspects of their work with colleagues: thus, of the 77% [n = 238; n = 111 AC (87.4%)] who stated that they were working with animals, 18.5% (21.6% AC) had had frequent, 67.5% (71% AC) occasional, and 14% (7% AC) no such discussions. Even among the respondents stating that they never had ethical concerns about their work, the majority indicated that they had discussed ethical aspects of animal use with peers at least occasionally (66.7% BC and 83% AC) (Fig 3).

Course participants were asked, first, how often they had ethical doubts or concerns about animal use (bold-outlined bars with percentage labels), and then how often these concerns were discussed with colleagues (excluding discussions for the purpose of project evaluation). A distribution of answers to the second question is presented for each subset of answers to the first question, as coloured stacked columns (legend on the bottom-right). Participants answering ‘I have not yet carried out animal experiments’ (72/310 BC and 16/127 AC) were excluded from this analysis.

### 3.3. Awareness of the 3Rs

**Fig 4:**
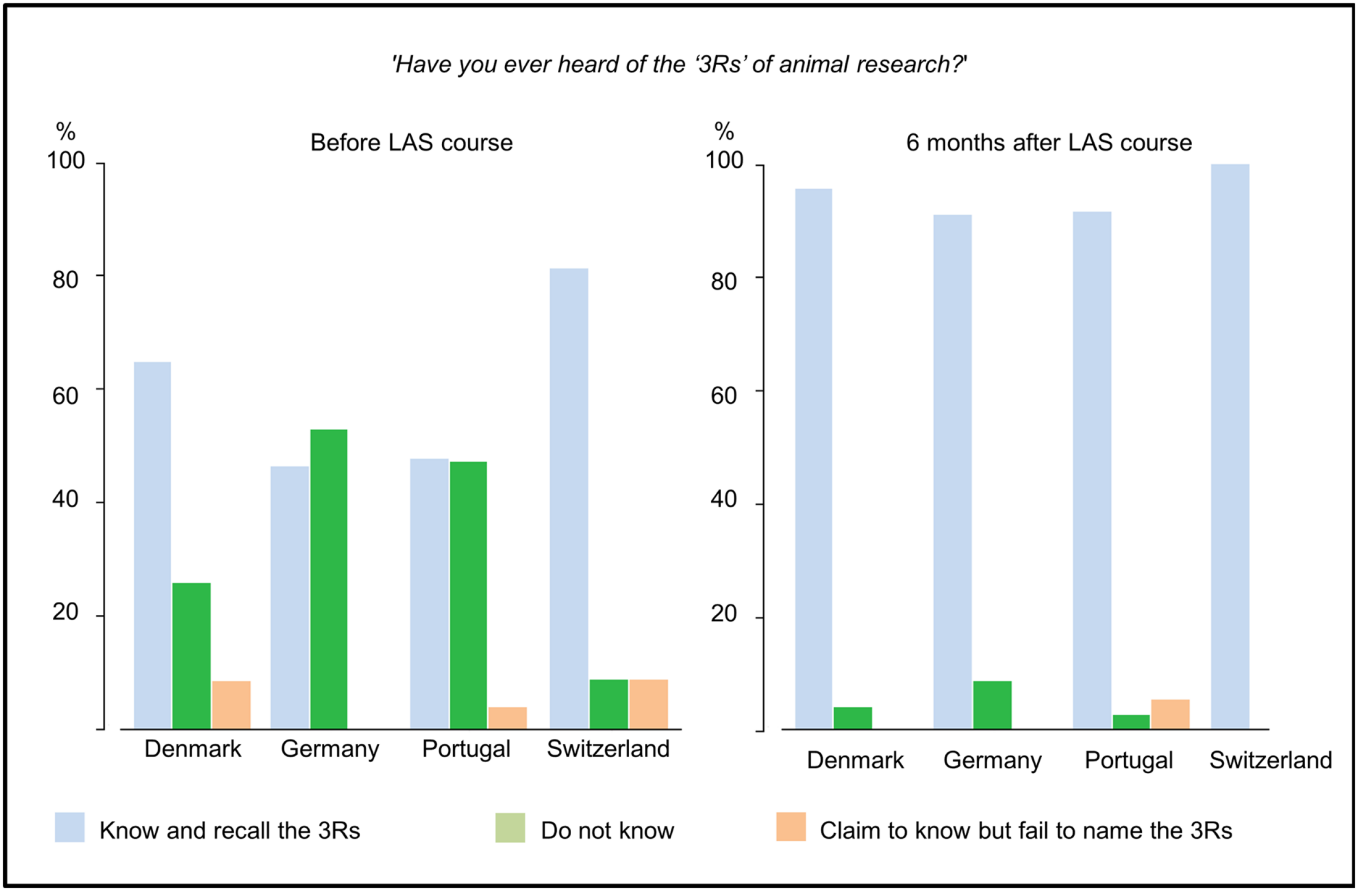
Awareness of the 3Rs. Respondents were asked to reply ‘Yes’ or ‘No’ to the question ‘Have you ever heard of the “3Rs” of animal research?’. Those answering ‘Yes’ were prompted to name the 3Rs.

Respondents were asked if they had heard of the 3Rs, and if they answered affirmatively they were promptly asked to name them. The majority (52.9%) were able to name the 3Rs correctly at the start of the course, 42.3% said they did not know of these principles, and 4.8% claimed to know the 3Rs but failed to name them correctly. BC awareness of the 3Rs varied significantly between countries (χ^2^ = 13.336; p = 0.004). Awareness also increased with years of experience with laboratory animals (χ^2^ = 24,127; p = 0.002; linear-by-linear association p<0.001), ranging from 37.7% in those with no experience to 87.5% in those with more than ten years of experience. Such differences disappeared in the follow-up survey (Fig 4). By that stage, a large majority of respondents (92.9%) could name all three of the 3Rs.

### 3.4. Attitudes to the 3Rs

Attitudes to the 3Rs were assessed using Likert-scale responses to a range of statements. The first set of statements expressed general attitudes to the 3Rs. The remaining statements focused on individual principles or prompted respondents to compare one principle with another. Table 1 (N = 233 BC; N = 98 AC) presents responses to these questions in both the first survey and the follow-up. In both surveys, no statistically significant differences between male and female researchers (Mann-Whitney test, with ‘not sure’ responses treated as missing values) for any of the statements were found.

Most respondents felt their research already showed sufficient consideration for the 3Rs, but interestingly a majority also stated that they intended to implement the 3Rs further. Self-assessed conversance with the 3Rs also rose between the two surveys, with agreement (‘agree’ plus ‘strongly agree’) with the statement ‘I do not know about the 3Rs as much as I want to’ dropping from 46.8% before the course to 13.3% six months later (no statistical test carried out, since the data were unpaired).

Just over half of respondents agreed (56.5% BC, 51.0% AC) that ‘Any animal experiments are acceptable provided the 3Rs are fully considered’. More than two thirds, before and after the course, disagreed with the statement ‘I have no issues with relevant and scientifically sound animal experiments, even if the 3Rs are not fully considered’.

A substantial majority of respondents (72.1% BC, 80.4% AC) agreed that animal Replacement is contingent on scientific and technological developments. The statement ‘Non-animal methods have their own place and value in biomedical research, and should not be seen as mere alternatives to animal experiments’ also received considerable support (70.4% BC, 65.0% AC). Turning to other factors that could affect decisions on Replacement, most respondents (53.7% BC, 63.9% AC) agreed that results from animal studies make it easier to publish in high-ranking journals. Only a small proportion (4.8% BC, 7.2% AC) agreed that replacing animals in their own research would be too expensive.

The potential of non-animal methods to replace animal experimentation was assessed by respondents as low: 67.0% (76.3% AC) disagreed that ‘full Replacement’ is achievable in the foreseeable future. It was agreed by a large proportion of respondents (81.7% BC, 77.3% AC) that there is room for some Replacement, but that animal experiments will always be necessary. Full Replacement was also considered less ‘readily achievable’ (80.0% BC, 87.6% AC) and less ‘urgent’ (62.4% BC, 70.1% AC) than full Refinement. Further support for Refinement was shown by the numbers of respondents who disagreed with the idea that Refinement might interfere negatively with the reproducibility of animal experiments (51.7% BC, 62.2% AC) and who agreed that *how* animals are treated is more important than *how many* animals are used (53.7% BC, 55.1% AC). The strongest consensus was found for the statement ‘Refinement measures are a prerequisite for the quality of animal research’ (88.5% (94.9% AC) agreed).

**Fig 5:**
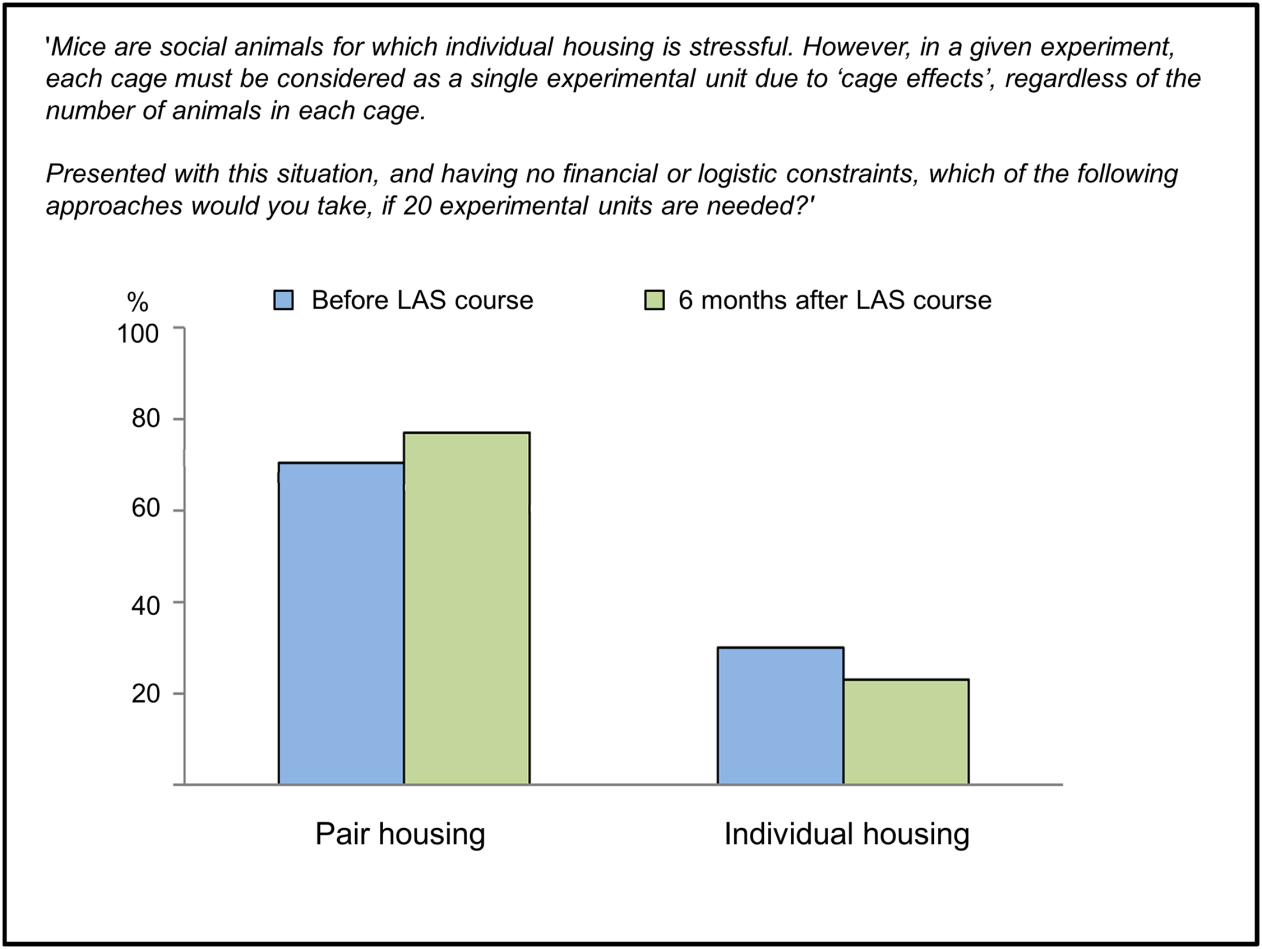
Responses to a Refinement-Reduction dilemma. The Refinement was to house animals in groups, potentially conflicting with the principle of Reduction when a treatment is administered at cage-level and the cage can therefore count as only one experimental unit. (N = 233 BC, N = 97 AC).

Refinement measures are important in ethically sound animal research, but they can sometimes come into conflict with Reduction or with scientific priorities [11,14,15,30]. Four hypothetical case studies were thus presented to assess attitudes and values relating to Refinement, and beliefs about the relationship between Refinement and Reduction, as well as the weight that should be given to these principles when they come into conflict with experimental design. The first involved a Reduction-Refinement dilemma. Respondents were required to choose between pair housing or single housing of mice, where the first option had known welfare benefits for mice, being social animals, but required twice as many animals to remain statistically powerful. The responses (Fig 5) confirm that the vast majority of course participants gave priority to Refinement over Reduction and preferred to use more animals rather than housing the animals in a way that does not cater to their social needs. In both surveys no significant country, gender or age differences were found.

In the second case study respondents were asked to decide whether to add nesting material to the cages of mice modelling a neurodegenerative disease as an environmental enrichment, knowing that this might affect the phenotype. When one of the two options (standard or enriched housing) was selected, a set of further options appeared that were designed to explore the reasons behind the decision (Table 2). In both surveys no significant country, gender or age differences were found.

**Table 2:** Standard housing vs. environmental enrichment (N = 228 BC, N = 96 AC). This dilemma was based on the known impact of nesting material on the phenotype of animal models of neurodegeneration, and on the legal requirement to provide environmental enrichment. For each of the two sub-groups (i.e. those preferring standard housing and those favouring environmental enrichment) a list of possible justifications was provided. Respondents were asked to choose from (one or more) or these, or to select an open-ended ‘other’ option. Values in the table are percentages of respondents choosing each justification within the relevant sub-group of respondents.

“*Environmental enrichment (EE)—e*.*g*. *providing nesting material—is broadly regarded as an important Refinement for laboratory rodents*. *However*, *in transgenic mouse models of Huntington’s disease*, *EE significantly delays the onset of disease*, *slows its rate of progression and extends survival time considerably*. *If you were to use these animal models*, *how would you choose to house them*? “					
BC %	Standard housing (42.8% BC, 46.9% AC)	AC %	BC %	Environmental Enrichment (57.2% BC, 53.1% AC)	AC %
37.8	EE causes undesired variability between groups	46.7	65.6	EE does not confound results if provided to both experimental and control groups	74.5
58.2	The therapeutic effect of EE is a confounding factor, masking treatment efficacy and skewing results.	62.2	22.1	Treatment efficacy must be higher than therapeutic effect of EE.	25.5
38.8	Standard housing allows comparability of results with other labs.	37.8	16.0	It is impossible to replicate the exact setting from lab to lab. If results are robust, they will be reproducible in slightly varying settings.	29.4
28.6	Benefits of EE are outweighed by the cost of its interference with results.	20.0	20.6	The benefits of EE outweigh any potential variance in results	33.3
31.6	The added variability of EE requires using more animals.	53.3	13	If EE is confirmed to add variability (i.e. in a pilot), this can be balanced by increasing ‘n’ per group and improving experimental design	27.5
54.1	Unreliable data from mice housed with EE may lead to wasting animals’ lives and resources.	62.2	32.8	Unreliable data from standard housed mice may lead to wasting animals’ lives and resources.	41.20
9.2	Cage bedding and ad lib water and food are sufficient to supply animals’ basic needs, while EE unnaturally extends survival	8.9	48.9	Animals in non-enriched cages show an unnaturally accelerated phenotype as a result of sensorial deprivation.	52.9
7.1	Other	6.7	3.8	Other	9.8

The respondents showed a slight preference for environmental enrichment, both before and after the course, but this was not significant for our chosen alpha (χ^2^ = 4.755 p = 0.029 BC; χ^2^ = 0.375, p = 0.54 AC). Most respondents with this preference justified it by agreeing that enrichment does not confound results provided that it is given to all animals, and by agreeing also that animals in non-enriched cages show an unnaturally accelerated phenotype as a result of sensorial deprivation. The most common concerns of those who chose standard housing were the potential of enrichment to become a confounding factor and the waste of animals that would result from unreliable research. Concern about the need to use more animals to deal with the added variability that would result after environmental enrichment was higher in the follow-up survey.

**Fig 6:**
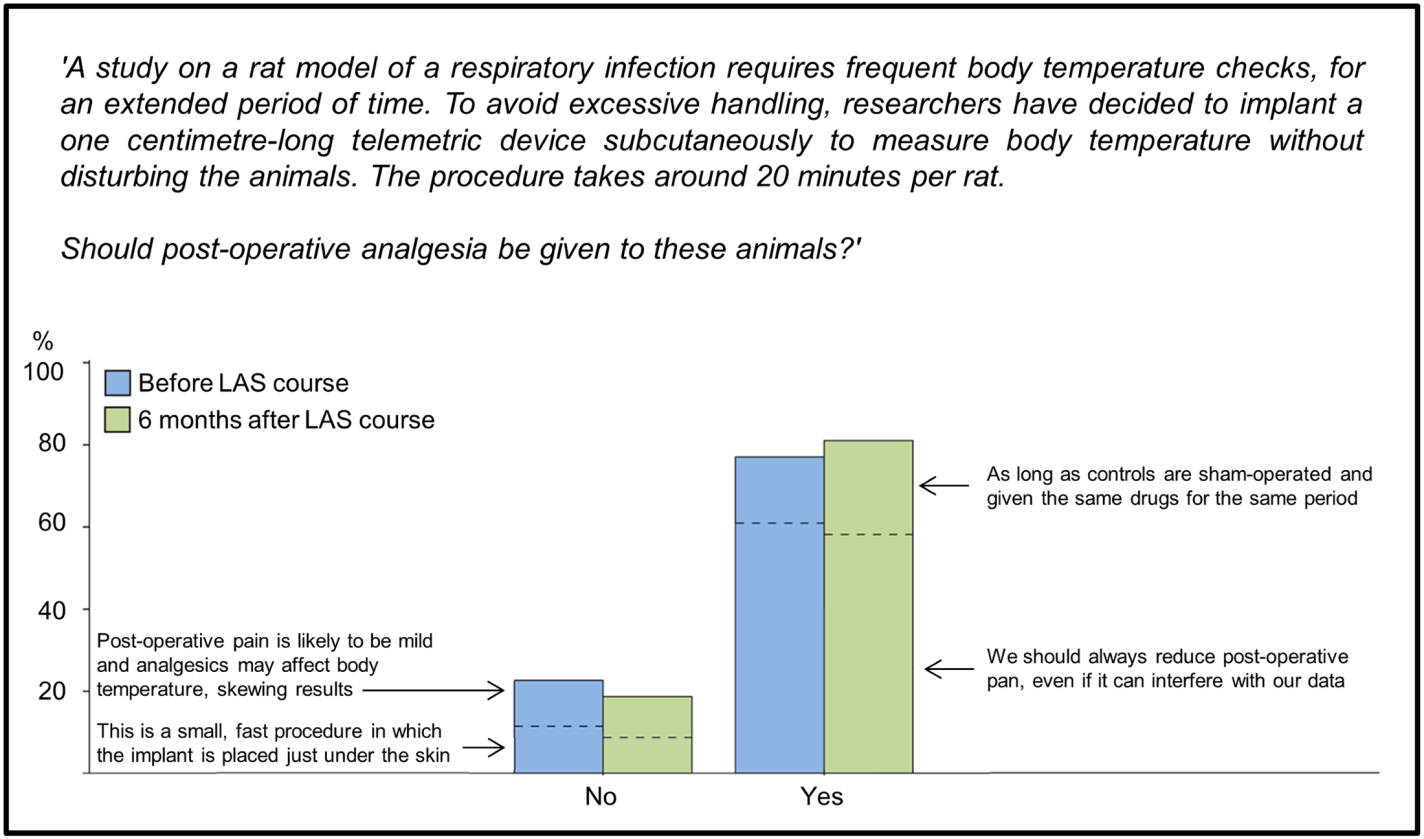
Case study: Provision of analgesia to rats. Respondents were told that a Refinement measure, post-operative analgesia, could potentially affect a parameter of interest, i.e. body temperature. Four options were available: two for ‘yes’ and two for ‘no’. The ‘yes’ and ‘no’ pairs appear stacked together in the bar chart. The statements on the left and right explain the ‘no’ and ‘yes’ options. (N = 223 BC, N = 91 AC).

In the third case study, respondents were asked whether post-operative analgesia should be given to rats following a small surgical procedure to implant a transmitter in them subcutaneously. In both surveys, most respondents (77.1% BC, 81.3% AC) indicated that they would provide post-surgical analgesia, and the majority of these (61.0% BC, 58.2% AC of the overall sample, Fig 6) also preferred to provide it in sham-operated controls. Only a small proportion (16.1% BC, 23.1% AC of the overall sample) considered that pain management should always be carried out, even if it interfered with data. In both surveys no significant country, gender or age differences were found.

**Fig 7:**
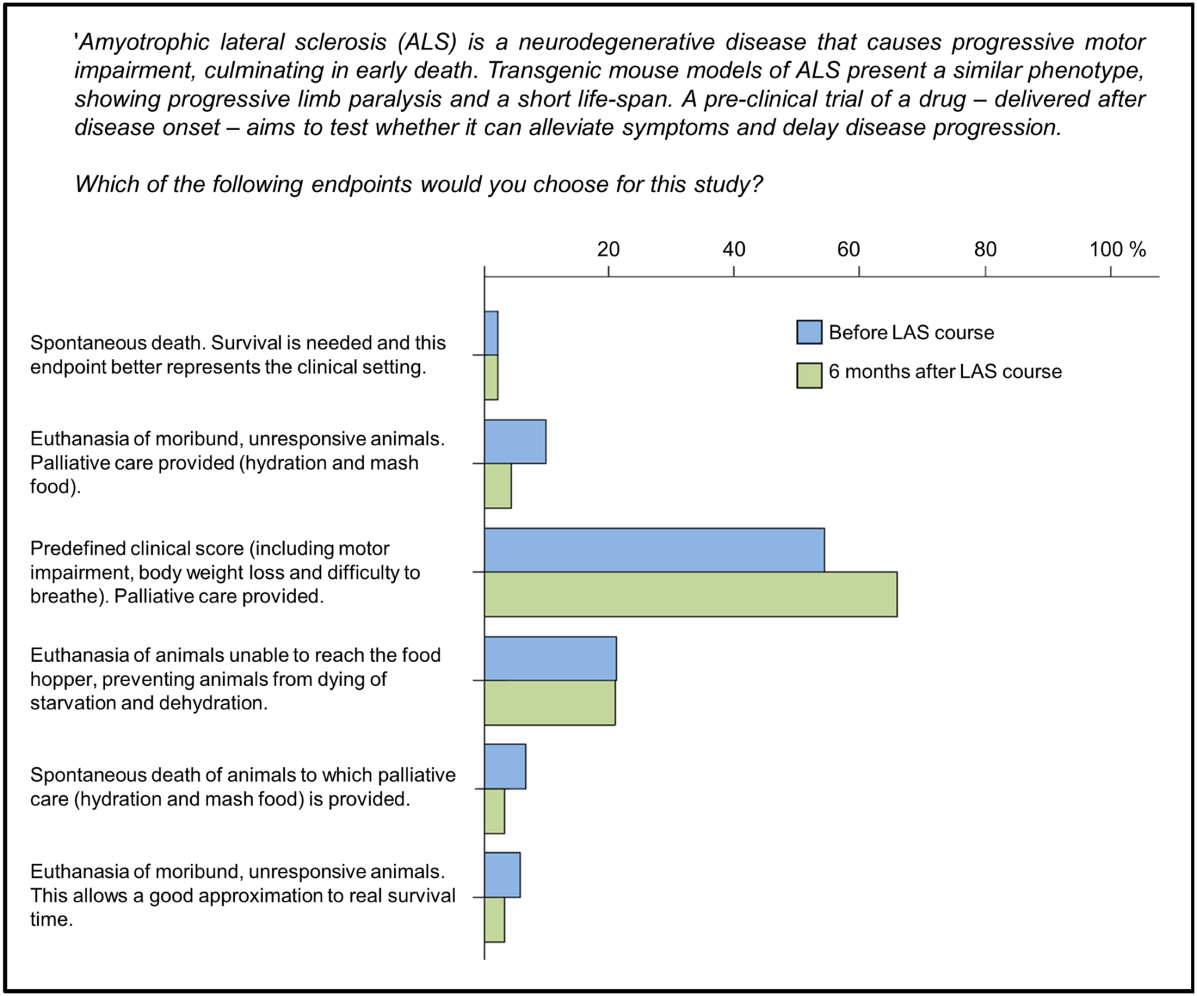
Case study on humane endpoints. Respondents were asked to choose one of several possible endpoints (including an open-ended ‘other’ option) for an efficacy test of a drug to treat Amyotrophic Lateral Sclerosis (ALS). (N = 223 BC, N = 91 AC).

In the fourth case study, respondents were asked to consider the definition of a humane end-point for studies using a murine model of Amyotrophic Lateral Sclerosis (ALS) (Fig 7). There was very little support for spontaneous death as an endpoint, or for very late-stage endpoints. A scoring system based on clinical signs (54.3% BC, 65.9% AC) and euthanasia of animals when motor impairment would prevent them from reaching food and water (21.1% BC, 20.9% AC) were the preferred options. In both surveys no significant country, gender or age differences were found.

### 3.5. Self-assessed learning and the impact of LAS topics

**Fig 8:**
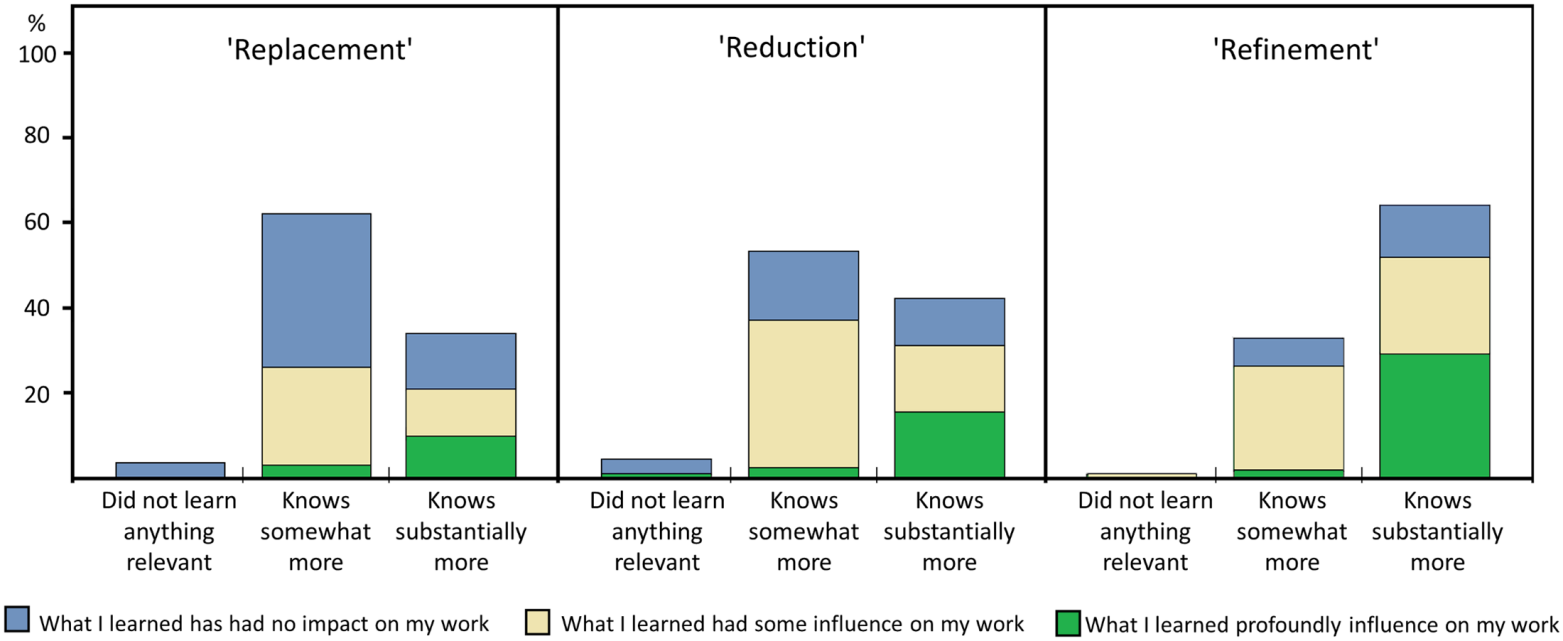
Self-assessed learning about the 3Rs (in follow-up survey, N = 90). Original response options: ‘I did not learn anything of relevance on this topic’; ‘I know somewhat more than I did, from what I learned in the course’; ‘I know substantially more than I did, from what I learned in the course’. For each item (described as ‘Replacement alternatives to animal experiments’; ‘Reduction of the number of animals used for each experiment’; and ‘Refinement of procedures to minimize harm and improve wellbeing’) respondents were prompted to indicate the impact of the taught content in their work (stacked coloured bars, items originally described as ‘What I learned has had no impact on my current work’; ‘What I learned on this topic had some influence on my work with animals’; ‘What I learned profoundly influenced my work with animals’).

In the follow-up survey participants were asked how much they had learned about a range of topics covered in LAS courses (S2 Fig), including each of the 3Rs (Fig 8). Self-assessed levels of learning were higher for Refinement (Sign Test, p<0.001) than they were for Replacement and Reduction (with no significant difference between the latter). Answers did not differ significantly with country, gender or type of course (FELASA B or C).

Participants in the follow-up survey were also asked about the impact of topics taught on LAS courses on their current work. Among the topics listed, ‘Replacement alternatives to animal experiments’ had the lowest self-reported impact (53.3% indicating it was not applied) as compared with all other topics (Sign test, p<.001). Of the three 3Rs, Refinement had the strongest impact on participants’ work (32.2% ‘profound influence’, 47.8% ‘some influence’), followed by Reduction (17.8% ‘profound influence’, 51.1% ‘some influence’) and Replacement (13.3% ‘profound influence’, 33.3% ‘some influence’) (Fig 8).

The specific topic with the highest levels of self-reported impact was ‘Handling techniques’ (45.6% ‘profound influence’, 38.9% ‘some influence’). The topics with lowest impacts were ‘Design of animal experiments’ and ‘Scientific validity and integrity of animal experiments’, which 21.1% and 25.6% of respondents, respectively, described as having had a ‘profound influence’ (S2 Fig).

## 4. Discussion

Mandatory training in laboratory animal science aims to raise researchers’ awareness of, and competence in applying the 3Rs. Researchers attending LAS training courses arrive, however, with their own values and assumptions, and this is bound to affect the way the intended learning outcomes of the courses (knowledge, attitudes and skills) are understood and ultimately applied.

Our first research question focused on the need for animal use and its ethical justification. Our results revealed very limited confidence in the idea that animal experimentation can be replaced by non-animal alternatives. We recognize the inherent bias in asking scientists seeking training to use animals whether they believe animal-use to be necessary, but this finding confirms a widespread—and for some, resolute [31,32]–view that full Replacement in biomedical research is widely viewed as impossible. Our results accord with previous surveys of scientists conducted in the UK [33], The Netherlands [34] and Canada [35], as well as a report from the University of Copenhagen on Danish researchers’ attitudes to the 3Rs [36].

Non-animal alternatives are more readily developed and validated in regulatory testing, e.g. in toxicology and vaccine assessments [37], than they are in basic and applied biomedical research [38,39,40], although many more animals are used in the latter [41]. It should be noted therefore that the respondents in our sample were more likely to be involved in biomedical research than regulatory testing, and that the scarcity of available Replacement alternatives in their own field of work could have affected their negative attitude to the feasibility of full Replacement.

Around half of the respondents took the 3Rs to provide a sufficient safeguard of laboratory animal welfare, agreeing that any animal experiment is acceptable provided the 3Rs are fully considered, and most rejected the idea that relevant and scientifically sound research is acceptable even where the 3Rs have not been applied. The prevailing view of the respondents appeared therefore to be that laboratory animal use should conform to the 3Rs and be relevant/sound. Like the general population, the researchers gave conditional approval to animal research [2,3,42].

Around three quarters of respondents acknowledged having at least occasional ethical concerns about their work, and considerably more indicated that they had discussed ethical issues with colleagues, going beyond regulatory requirements in doing so. This suggests that, despite their reluctance to agree that there were alternatives to animal use in their own research, the respondents recognized that animal experimentation does raise ethical issues. Researchers are often portrayed by animal rights groups as people with little or no regard for animal welfare. Our results help to explain why they feel unjustly stigmatized by these groups [43,44]: while animal rights advocates tend to take a more uncompromising stance on animal welfare, our findings confirm that researchers have more nuanced, and sometimes conflicting, attitudes [45].

The second research question concerned researchers’ awareness of the 3Rs. More than half of our respondents were able to name the 3Rs before attending LAS training. Our data could not explain why this ability was distributed unevenly across the four countries, but since the disparities were not observed in the follow-up survey, we can say that the courses were effective in raising awareness of the 3Rs and rectifying national and institutional differences in researchers’ awareness of them. It was also encouraging to find that researchers with more experience of working with laboratory animals were more likely to be aware of the 3Rs before attending an LAS course.

In answering the third research question, about researchers’ attitudes to the 3Rs, we paid particular attention to prioritization. The respondents regarded full Refinement as both a higher priority and a more readily achievable goal than full Replacement. Non-animal methods were not dismissed, however. Two thirds agreed that these have a place, are valuable in biomedical research, and should not be seen as mere alternatives to animal methods. Moreover, most respondents denied that animal experimentation will only end when effective treatments are available for all human diseases, suggesting that there is a belief, among researchers, that full Replacement may at some point in the future be achievable. The respondents saw this as a technical challenge: a large majority indicated that full animal Replacement is contingent on the development of alternative methods.

Hypothetical cases were used to investigate researchers’ responses in situations where Refinement needs to be balanced against other Reduction and scientific requirements. In the case study involving mice the goals of Refinement (‘pair-housing’) and Reduction (‘single-housing’) were in direct conflict. Confirming our previous finding [46], we found that approximately two thirds of researchers prioritized Refinement and one third prioritized Reduction. This prioritization is interesting in ethical terms, as it suggests that the researchers were more concerned about the *level* of animal suffering than they were about *how many* animals suffered [46,47].

The remaining three case studies involved trade-offs between Refinement and scientific requirements. In the case study on humane endpoints in experimental infection research, early humane endpoints were preferred over later stage endpoints. The researchers chose to limit animal suffering even though this would mean that the study resembled what happens in the clinical setting less closely. The same prioritization was expressed in the next case study on whether to provide post-operative analgesia after surgically implanting a telemetry emitter: most respondents opted to relieve pain and deal with the potential impact on the scientific data by adapting the experimental design.

This last finding may appear to be inconsistent with a Canadian survey [35] in which only 9.7% of principal investigators and 14.2% of more junior researchers considered that ‘Pain relief should always be provided to animals during painful procedures’. However, the Canadian survey asked whether pain relief should *always* be given, which may have deterred respondents from responding with a firm ‘yes’. Our case study, by contrast, focused on a single experiment, and it was explained that potential impacts on scientific data could be dealt with by adapting the experimental design. It may also be relevant that the participants in our study were about to attend, or had attended, a course in which pain management is a prominent subject. It is also worth noting that the proportion of our respondents stating that pain relief should *always* be provided was very close to that found in the Canadian study for the sub-group of more junior researchers, and that this sub-group more closely resembled our own sample in terms of age, gender distribution and experience with laboratory animals.

In all four of our case studies the majority of respondents prioritized Refinement. This priority was less pronounced, however, in the nesting material vs. standard caging case. It is tempting to infer that sub-optimal housing conditions were regarded as a less serious ethical problem than pain (e.g. from surgery) or distress caused by social isolation. However, it should be noted that both the respondents who opted to provide nesting material *and* those who chose to use standard cages rationalized their decisions by referring to impacts on data variability. The former indicated that confounding effects could be addressed via experimental design. The latter were more concerned about the risk of skewed results. It is possible, therefore, that Refinement was selected less often in the nesting material vs. standard housing case than it was in the other hypothetical cases because respondents concluded that the use of nesting material raised more serious concerns about scientific validity.

The final research question asked how animal scientists rate the relevance and usefulness of LAS courses. We found that Replacement was the 3R principle course participants were least likely to say they ‘know substantially more’ about after attending the course. It was also the principle they reported applying least. Self-assessed knowledge gains were highest for Refinement, where they were confirmed for each of the listed techniques (handling, sampling, substance administration, anaesthesia and analgesia, and euthanasia and humane endpoints). Intermediate gains were reported for Reduction topics such as experimental design, and for scientific integrity and validity of animal experiments. Clearly, self-evaluations can reflect bias. Knowledge gains and their application in subsequent research may in reality be lower than respondents report. However, the self-reported knowledge gains and extent of application of each of the 3Rs found here were consistent with, and proportional to, their prominence in LAS courses syllabuses [23,24].

As for the representativity of the follow-up sample relative to the sample used in the original survey, the 60% attrition rate (after discarding respondents who did not recall answering the first survey) had no measurable effect on the demographic profile of the sample (e.g. mean age, gender, seniority, country, type of course) or on most of the results. Nor did adding the 73 respondents who had not replied to the first questionnaire. This suggests any bias arising from attrition was negligible. Several respondents failed to complete the survey, but this was not unexpected as the survey was voluntary and participants were free to withdraw at any point if they wished to do so.

It may be suggested that a survey of scientists undergoing mandatory training will not necessarily reflect attitudes across the laboratory animal science community as a whole. Against this, we would point to the diversity of our sample, in terms of nationality, age, gender, field of research, and experience with laboratory animals. We believe this diversity allows general trends to be tentatively inferred. The fact that the researchers we surveyed were at an early stage of their career may indeed be advantageous if it is future developments in animal laboratory science that we are interested in, since these respondents can be expected to be active for many years to come.

The fact that respondents from the BC and AC samples could not be paired meant that formal statistical comparison of inter-survey differences could not be conducted. However, separate analyses of the BC and AC data sets paint the same general picture, particularly where attitudes to the need for animal experiments and the potential of Replacement alternatives are concerned. Observable differences here related only to respondents’ reported experience of animal use and knowledge gains in certain areas (i.e. experimental design, availability of fish and invertebrate models, and awareness and self-reported knowledge of the 3Rs). Differences of the latter kind were further corroborated in the self-assessment of knowledge gains and the impact of 3Rs measures.

**Fig 9:**
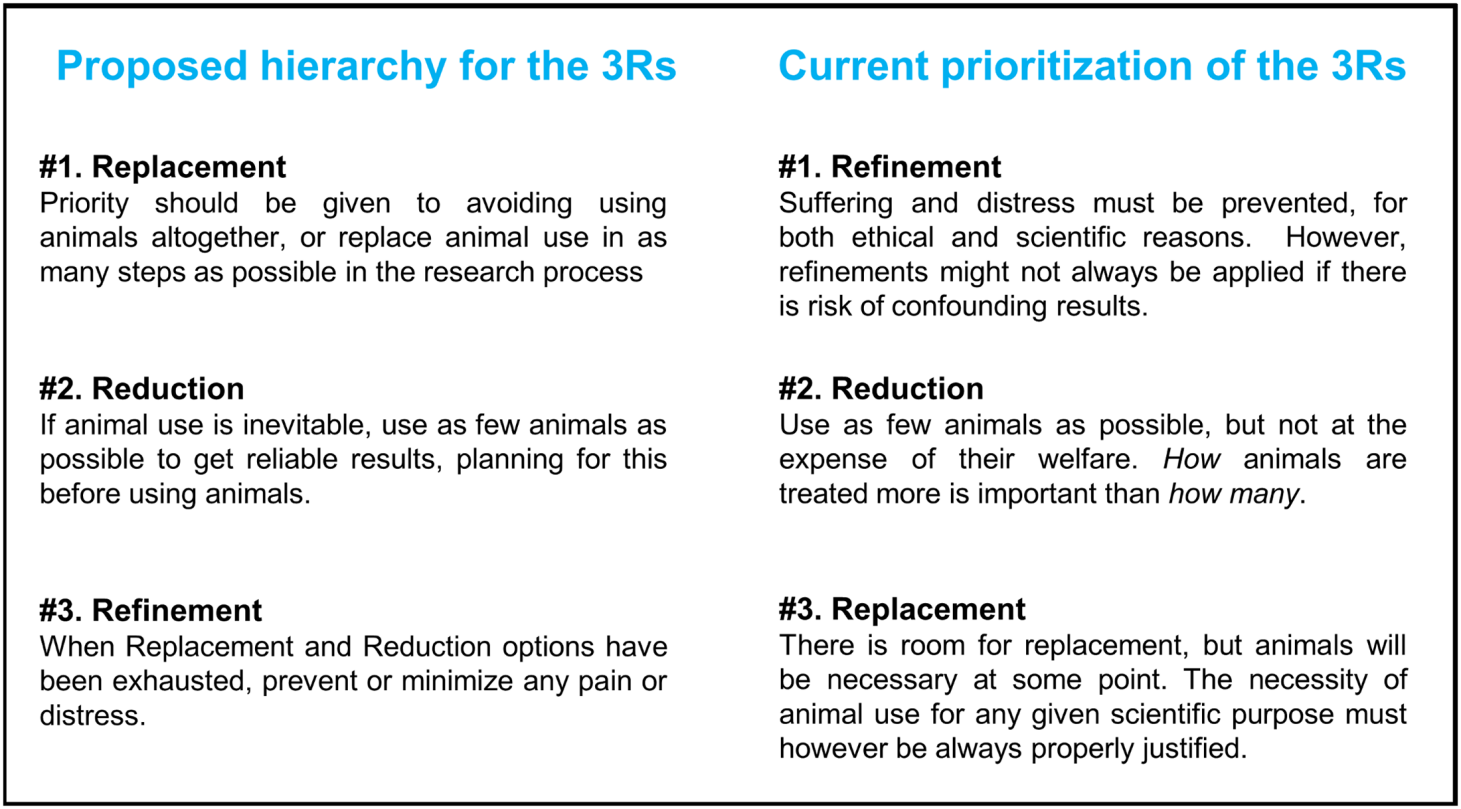
A comparison of two interpretations of the way scientists using (or intending to use) animals perceive the 3Rs. Summary descriptions of the principles were devised for this comparison.

In the classic exposition of the 3Rs given by Russell and Burch [5], the principles are set out in a hierarchy. Replacement—described as ‘always a satisfactory answer’–is the ultimate goal, and thus prioritized. Reduction precedes Refinement, because among other things Reduction strategies must be considered and established *before* using animals. Refinement is only to be applied after ensuring ‘Replacement is not (yet) possible, and every device of theory and practice to reduce the number of animals to a minimum has been employed’ [5]. In this sense, Russell and Burch regarded Refinement and Reduction as means to the proper end of ethical audit, Replacement [48]. The respondents in our sample reversed this ordering (as summarized in Fig 9). They appeared to start from the assumption that animal use is both necessary and inevitable, thus placing Replacement at the bottom of the list of priorities, a re-ordering that was also observed in a previous smaller survey of 48 Dutch scientists [34]. This is also consistent with the design of LAS courses, aimed at improving researchers’ knowledge and competence on the ethical use of laboratory animals, but not intended to improve knowledge and skills in non-animal methods, which has led to the proposal (e.g. by [34] and [49]) of dedicated training approaches to further the adoption and development of non-animal methods.

Animal research’s detractors may point to scientific conservativism and inertia, or even veiled financial, professional or political interests [50], as the main drivers of this upturned hierarchy. However, in a conference held in December 2016 by the European Commission [51] that gathered all major stakeholders—most notably animal protection associations, researchers in the public and private sectors, funders, regulators, and patient groups, among others—a quite different picture of animal research emerged, with scientists who in many ways resembled those in our sample. The conference revealed a scientific community that is sensitive to ethical issues and receptive to new non-animal research methods, but also aware of the size of the challenges facing those seeking to model the highly variable and complex biology of humans and other animals, along with its interactions with pathogens and the environment, particularly considering our still limited knowledge of these factors.

## 5. Conclusions

The following conclusions can be drawn from this survey. First, a picture emerges of scientists carrying out animal experiments as concerned professionals who certainly care about the ethical implications of their work, and who attach importance to both animal welfare and validity issues but are ultimately not convinced of the potential of alternative methods to fully replace animal use in their field. Both before and after the courses, in other words, Replacement was regarded with scepticism, with non-animal methods being mostly seen as complementary to animal use, or at best as potential alternatives to some steps in biomedical research.

In this regard, while LAS courses are effective in raising awareness and understanding of the 3Rs, they focus mostly on improving researchers’ knowledge and skills in Reduction and Refinement, thus having little impact on scientists’ understanding of, and readiness to use, methods in the life sciences in which animal use is avoided. We suggest that Replacement alternatives should remain a mandatory topic on the courses, but also that specific plans are needed to further the development and adoption of non-animal methods.

Second, most European researchers think about the ethics of their work and discuss the issues with peers, but while the overwhelming majority consider Refinement to be a pre-requisite of good research, most take the 3Rs to be already sufficiently implemented in their work, even prior to attending an LAS course.

Finally, the hierarchy of the 3Rs envisaged by Russell and Burch—with Replacement as the main goal, Reduction as an option when there are no non-animal alternatives, and Refinement as the last principle to be applied—appears to have been inverted by European scientists working today: they place Refinement first and Replacement last.

## Supporting information

S1 Fig Age of participants. Above, venue comparison for mean age of participants (first survey. error bars represent 95% confidence interval and asterisks indicate significant differences for p<0.01); below, age distribution of respondents in both surveys.
(TIF)

S2 Fig Self-assessed learning and its impact (eight topics, N = 90). Original response options: ‘I did not learn anything of relevance on this topic’; ‘I know somewhat more than I did, from what I learned in the course’; ‘I know substantially more than I did, from what I learned in the course’. Respondents were asked to assess the impact of each taught topic on their work (stacked coloured bars, items originally described as ‘What I learned has had no impact on my current work’; ‘What I learned on this topic had some influence on my work with animals’; ‘What I learned profoundly influenced my work with animals’).
(TIF)

S1 Table Sample characterization. Except in the category ‘experience with laboratory animals’ the demographics in the two surveys were similar.
(DOCX)

S1 Questionnaire Example Some questions depended on previous answers. These dependencies are explained. Some of the questions (Q7-Q13 of the second part) and their answers are not described in this paper and refer to an exercise with several ‘species-severity-purpose of research’ interactions. These are part of a separate study on the influence of such factors on the acceptability of animal research by scientists.
(PDF)

S1 Dataset Raw data SPSS file. (SAV)
