# Supplementary figures and images for: Researchers’ attitudes to the 3Rs—An upturned hierarchy?
Source: PLoS One. 2018 Aug 15;13(8):e0200895. doi: 10.1371/journal.pone.0200895 (PMC6093608; doi:10.1371/journal.pone.0200895)

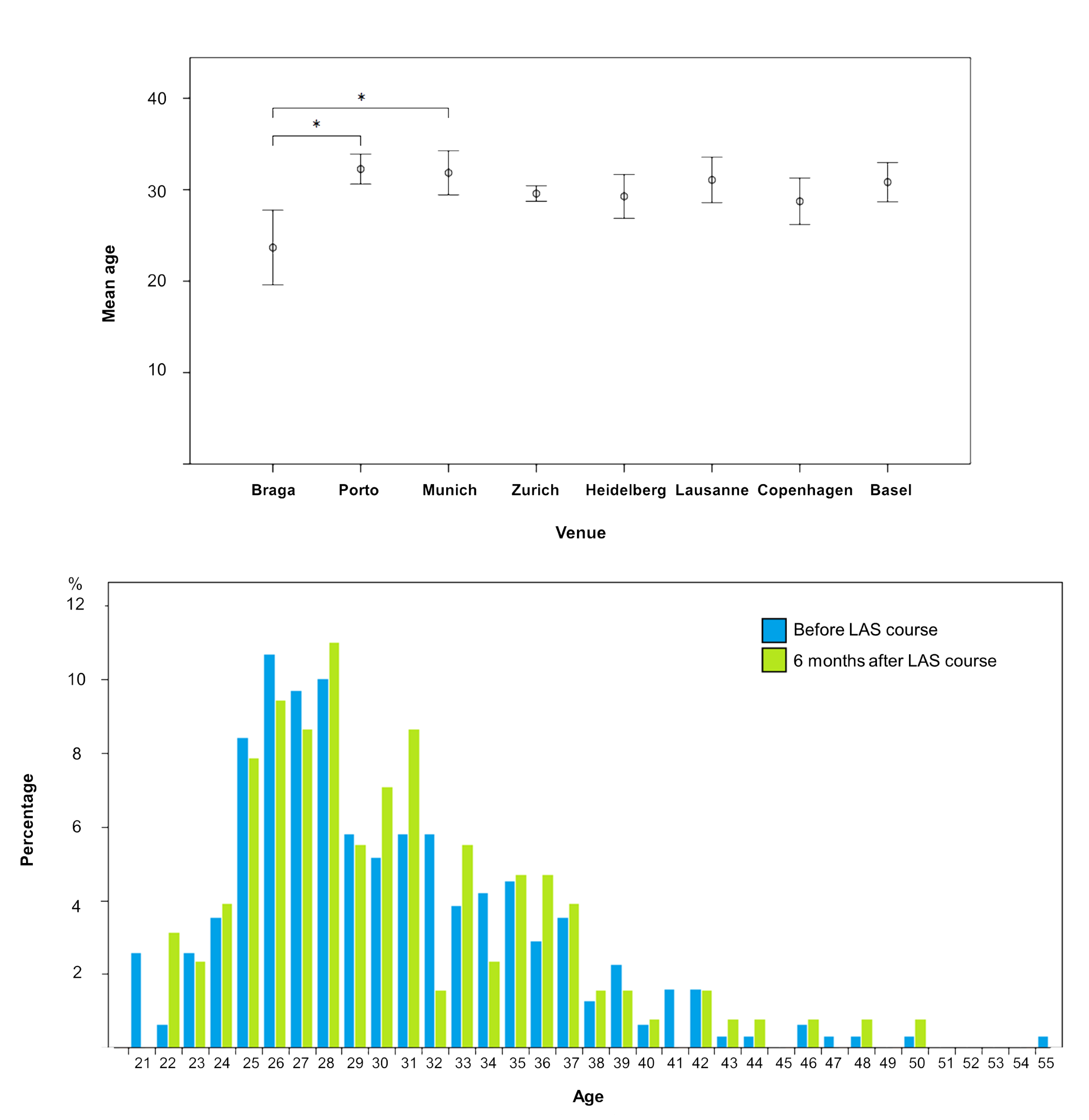

Supplement: S1 Fig — Above, venue comparison for mean age of participants (first survey. error bars represent 95% confidence interval and asterisks indicate significant differences for p<0.01); below, age distribution of respondents in both surveys. (TIF) [file pone.0200895.s001.tif]

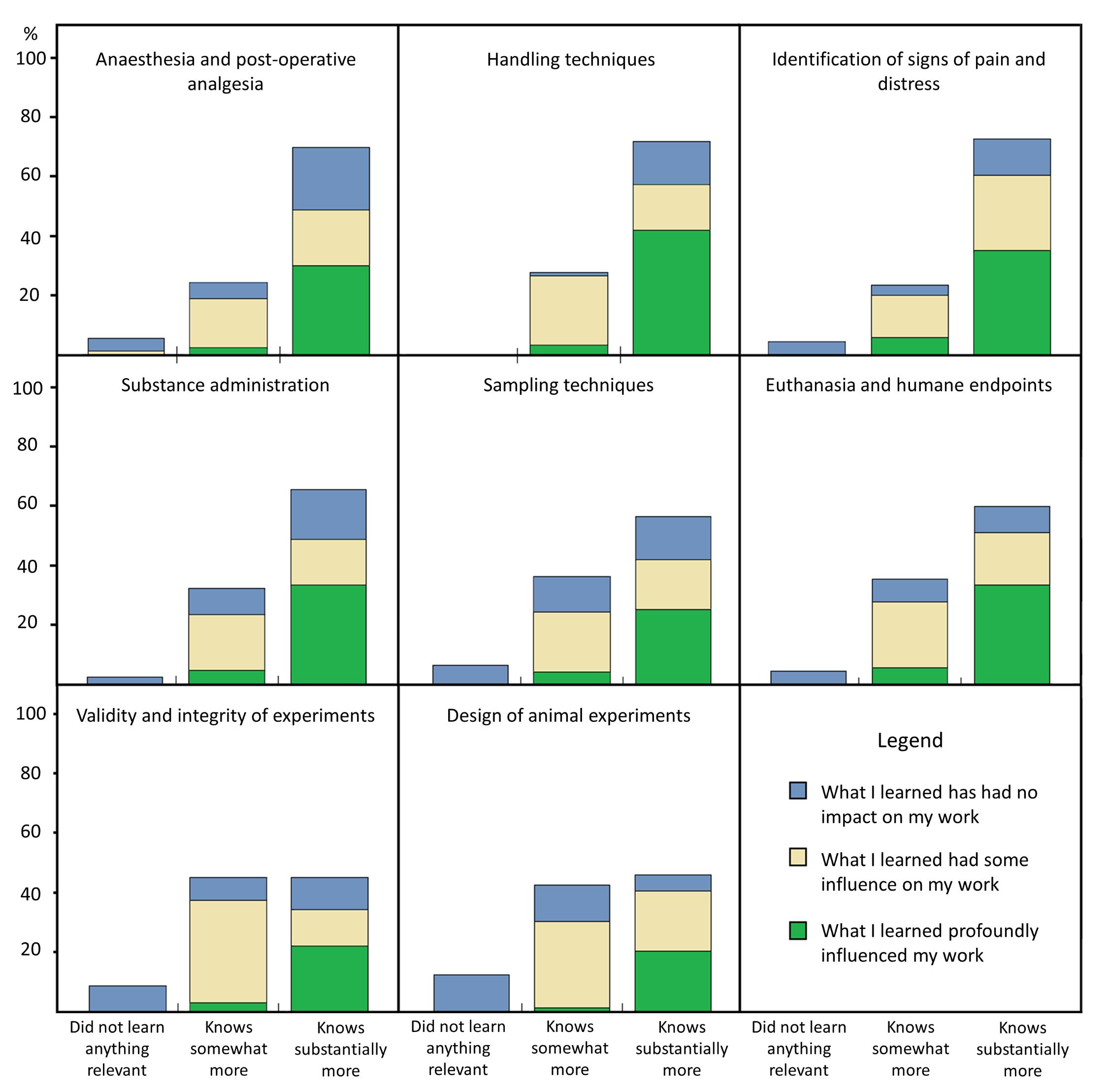

Supplement: S2 Fig — Original response options: ‘I did not learn anything of relevance on this topic’; ‘I know somewhat more than I did, from what I learned in the course’; ‘I know substantially more than I did, from what I learned in the course’. Respondents were asked to assess the impact of each taught topic on their work (stacked coloured bars, items originally described as ‘What I learned has had no impact on my current work’; ‘What I learned on this topic had some influence on my work with animals’; ‘What I learned profoundly influenced my work with animals’). (TIF) [file pone.0200895.s002.tif]
