# Supplementary material for: Researchers’ attitudes to the 3Rs—An upturned hierarchy?
Source: PLoS One. 2018 Aug 15;13(8):e0200895. doi: 10.1371/journal.pone.0200895 (PMC6093608; doi:10.1371/journal.pone.0200895)
Supplement: S1 Table — Except in the category ‘experience with laboratory animals’ the demographics in the two surveys were similar. (DOCX) [file pone.0200895.s003.docx]

|  | First Survey | Follow-up |
| --- | --- | --- |
| Course type | 70.0% ‘FELASA B’  30.0% ‘FELASA C’ | 71.4% ‘FELASA B’  28,6% FELASA C’ |
| Country  (were courses were held) | Denmark - 4%  Germany – 15%  Portugal – 22%  Switzerland – 60% | Denmark - 6%  Germany – 17%  Portugal – 19%  Switzerland – 58% |
| Gender | Female – 66%  Male – 35% | Female – 59%  Male – 41% |
| Occupation | Lab Technician – 7%  BSc/MSc student – 12%  PhD student – 41%  Postdoc – 27%  Faculty/PI – 5%  Other* – 8%  *mostly MDs or medical students | Lab Technician – 7,9%  BSc/MSc student – 8,7%  PhD student – 46,0%  Postdoc – 24,6%  Faculty/PI – 5,6%  Other* – 7,1%  *mostly MDs or medical students |
| Topic of first degree | Biology – 50.5%  Biochemistry – 13.6%  Pharmaceutical Science–6.1%  Medicine – 8.7%  Veterinary Medicine – 5.5%  Other – 15.5% | Biology – 49,4%  Biochemistry – 14,3%  Pharmaceutical Science–3,2%  Medicine – 10.3%  Veterinary Medicine – 6.3%  Other –16.5% |
| Topic of Ph.D. | Biology – 49.6%  Biochemistry – 9.4%  Pharmaceutical Science–5.4%  Medicine – 11.6%  Veterinary Medicine – 2.7%  Other – 21.3% | Biology - 45,8%  Biochemistry - 7,3%  Pharmaceutical Science -5,2%  Medicine 11,5%  Veterinary Medicine - 3,1%  Other - 27,1% |
| Experience with laboratory animals | None – 36.8%  Less than one year – 30.3%  1-5 years – 21.9%  6-10 years – 8.4%  >10 years – 2.6% | None – 17,5%  Less than one year – 38,1%  1-5 years – 33,3%  6-10 years – 7,1%  >10 years – 4,0% |
| Support of animal protection associations | No – 66.5%  Paying member – 5.2%  Active member – 0.3%  Has given donations –5.5%  Just sympathizer –22.6% | No – 68,3%  Paying member – 6,3%  Active member – 0,8%  Has given donations – 6,3%  Just sympathizer – 18,3% |

**S1 Table. Sample characterization.** Except in the category ‘experience with laboratory animals’ the demographics in the two surveys were similar.
