# Supplementary material for: Researchers’ attitudes to the 3Rs—An upturned hierarchy?
Source: PLoS One. 2018 Aug 15;13(8):e0200895. doi: 10.1371/journal.pone.0200895 (PMC6093608; doi:10.1371/journal.pone.0200895)
Supplement: S1 Questionnaire Example — These dependencies are explained. Some of the questions (Q7-Q13 of the second part) and their answers are not described in this paper and refer to an exercise with several ‘species-severity-purpose of research’ interactions. These are part of a separate study on the influence of such factors on the acceptability of animal research by scientists. (PDF) [file pone.0200895.s004.pdf]

Welcome to our Laboratory Animal Science follow-up questionnaire.

The questionnaire is divided in two parts:

- The first part takes about 1-3 minutes.
- The second part takes about 15-20 minutes.

The anonymity of respondents will be rigorously respected. **Please, answer sincerely.**

Please do not forget to proceed to the second part after completion of this first part
(by following the link provided after completion of this part).
There are 17 questions in this survey.

---

1. Before you start, please answer this question:

Did you fill in a similar questionnaire when you attended a course in laboratory animal science 6 months ago? *

Please choose only one of the following:

- ○ Yes
- ○ No
- ○ I don't remember

2. Please indicate your year of birth *
Only numbers may be entered in this field. Your answer must be between 1900 and 2000

Please write your answer here: [ ]

3. Sex *
Please choose only one of the following:

- ○ Female
- ○ Male

4. Nationality *

Please choose only one of the following:

- ○ [Country where the survey was being carried out]
- ○ Other [ ]

5. Main occupation *

Please choose only one of the following:

- ○ Lab technician
- ○ Bachelor`s/Master`s student
- ○ PhD student
- ○ Post-doc
- ○ Faculty Teacher/P.I.
- ○ Other

6. Select which options better describe the field in which you got your first degree *

Please choose only one of the following:

- ○ Biology
- ○ Biochemistry
- ○ Biophysics
- ○ Chemistry
- ○ Pharmaceutical sciences
- ○ Physics
- ○ Medicine
- ○ Veterinary Medicine
- ○ Other

7. Please select which option better describes the field in which you got your postgraduate degree (PhD) * [Only answer this question if the following conditions are met: Answer was 'PhD student ' *or* 'Post-doc ' *or* 'Faculty Teacher/P.I. ' at question '5 [Q0004]' (Main occupation)]

Please choose only one of the following:

- ○ Biology
- ○ Biochemistry
- ○ Biophysics
- ○ Chemistry
- ○ Pharmaceutical sciences
- ○ Physics
- ○ Medicine
- ○ Veterinary Medicine
- ○ Other

8. Please select which option(s) better describe your line of research, or scientific interests, if applicable. *

Please choose all that apply:

- ○ Understanding of basic biological mechanisms
- ○ Understanding of disease mechanisms for application in human medicine
- ○ Understanding of disease mechanisms for application in veterinary medicine
- ○ Development of disease treatments application in human medicine
- ○ Development of disease treatments for application in veterinary medicine
- ○ Other:

9. What is your experience with laboratory animals? *

Please choose only one of the following:

- ○ None
- ○ < 1 year
- ○ 1-5 years
- ○ 6-10 years
- ○ >10 years
- ○ Other

10. Which course did you attend? *

Please choose **only one** of the following:

- ○ Following FELASA recommendations for Category B
- ○ Following FELASA recommendations for Category C

11. Have you ever supported any animal rights or animal protection association? *

Please choose only one of the following:

- ○ No
- ○ As a paying member
- ○ As an active member
- ○ I’ve given donations
- ○ I just sympathize with the cause

12. How would you classify the relevance of animal experimentation <u>in your own scientific work</u>? *

Please choose **only one** of the following:

- ○ It is a central part of my work. Without it, my scientific activity would practically stop.
- ○ It is an important part of my work. Without it my scientific activity would be considerably affected
- ○ I mostly use non-animal methods, but my research does require some animal experimentation.
- ○ I don’t use live animals, only animal-derived materials (cell cultures, tissue samples, Foetal Bovine Serum, etc.)
- ○ My work does not require animal experimentation or the use of any product of animal origin.
- ○ I haven’t performed any animal studies yet, but I intend to in the near future.

13 Is there any step for which you are presently using animals and for which you see a potential for using alternative (non-live animal) methods? *

Please choose only one of the following:

- ○ No
- ○ Yes
- ○ I haven't used animals in my experiments yet

14. <u>How often</u> do you have any ethical doubts or concerns regarding animal use <u>in your own work</u>? *

Please choose only one of the following:

- ○ Frequently
- ○ Occasionally
- ○ Never
- ○ I haven't performed animal experiments, so far

15 How often do you <u>discuss ethical aspects</u> of your work with animals with colleagues (aside those elicited by regulatory demands)? *

Please choose only one of the following:

- ○ Frequently
- ○ Occasionally
- ○ Never
- ○ I haven't performed animals experiments, so far

16 Have you ever heard of the “3 Rs” of animal research? *

Please choose only one of the following:

- ○ Yes
- ○ No

17 Please identify the Three Rs. *

[Only answer this question if the following conditions are met: Answer was 'Yes' at question '16 [Q0015]' (Have you ever heard of the “3 Rs” of animal research?)]

Please write your answer here:

Submit your survey.

Thank you for completing this survey.

You have successfully completed the FIRST PART of our questionnaire, thank you.

Now <u>please proceed TO THE SECOND PART</u>, following the link below:

Welcome to the Second Part of the Laboratory Animal Science follow-up questionnaire

---

This part will take about 15-20 minutes.

The anonymity of respondents will be rigorously respected. **Please, answer sincerely.**

There are 20 questions in this survey.

---

1 **Regarding the ‘Three Rs’, please rate your degree of agreement with the following statements:** *
Please choose the appropriate response for each item:

	Strongly disagree	Disagree	Neither agree nor disagree	Agree	Strongly agree	I’m not sure
'Refinement' measures are a prerequisite for the quality of animal research	○	○	○	○	○	○
My research protocols have sufficient consideration for the 3 Rs	○	○	○	○	○	○
I intend to further implement the 3 Rs in my work	○	○	○	○	○	○
I do not know about the 3 Rs as much as I want to	○	○	○	○	○	○
'Refinement' measures can negatively interfere with the reproducibility of animal experiments	○	○	○	○	○	○
How animals are treated is more important than how many animals are used	○	○	○	○	○	○
I find any animal experiment acceptable, provided the 3 Rs are fully considered	○	○	○	○	○	○
I have no issues with relevant and scientifically sound animal experiments, even if the 3 Rs are not fully considered	○	○	○	○	○	○

2. **Please read the following case-study:**

Mice are social animals, for which individual housing is stressful. However, in a given experiment, each cage must be considered as a single experimental unit due to “cage-effects”, regardless of the number of animals in each cage.

Presented with this situation, and having no financial or logistic constraints, which of the following approaches would you take, if 20 experimental units are needed? *

Please choose only one of the following:

- O Pair housing, N=40 mice.
- O Individual housing, N=20 mice

3 **Regarding ‘Replacement’, please select your level of agreement with the following statements:** *
Please choose the appropriate response for each item:

	Strongly disagree	Disagree	Neither agree nor disagree	Agree	Strongly agree	I’m not sure
Full replacement of animal experimentation can be achieved in the foreseeable future	O	O	O	O	O	O
There is room for some replacement but ultimately, animal experiments will always be necessary	O	O	O	O	O	O
Non-animal methods have their own place and value in biomedical research, and should not be seen as mere alternatives to animal experiments	O	O	O	O	O	O
The end to animal experiments will only be possible when effective treatments are available for all known diseases	O	O	O	O	O	O
The most effective step to reduce the number of animals used would be to apply more rigorous criteria regarding which projects merit approval	O	O	O	O	O	O
Full ‘Refinement’ of animal experiments is more urgently needed than their replacement	O	O	O	O	O	O
Whether animal experiments can be replaced or not will depend on the level of scientific and technological development of alternative methods	O	O	O	O	O	O

	**Strongly disagree**	**Disagree**	**Neither agree nor disagree**	**Agree**	**Strongly agree**	**I’m not sure**
Full ‘Refinement’ of animal experiments is a more readily achievable goal than full ‘Replacement’	○	○	○	○	○	○
Having results from animal studies makes it easier to publish research in a high-ranking journal	○	○	○	○	○	○
Using fish, rather than mammals (such as mice) is a relevant 'Replacement'	○	○	○	○	○	○
In my case, replacing animal experiments for non-animal alternatives would be too expensive	○	○	○	○	○	○
Using invertebrates (other than cephalopods, e.g. nematodes or arthropods), rather than vertebrate animals, constitutes relevant 'Replacement'	○	○	○	○	○	○

4. **<u>Please read the following case-study</u>:**

> *Environmental enrichment (EE) – e.g. providing nesting material – is broadly regarded as an important refinement for laboratory rodents. However, in transgenic mouse models of Huntington’s disease, EE significantly delays the onset of disease, slows its rate of progression and extends survival time considerably.*

If you were to use these animal models, how would you choose to house them? Please select the option that best suites your view. *

Please choose only one of the following:

- ○ Standard housing
- ○ Providing environmental enrichment to animals

5. Please select the main reason(s) justifying your choice: [Only answer this question if the following conditions are met: Answer was “Standard Housing”. ' at question '4 [Q0004]'**]***

Please choose all that apply:

- ☐ EE causes undesired variability between groups
- ☐ The therapeutic effect of EE is a confounding factor, masking treatment efficacy and skewing results.
- ☐ Standard housing allows comparability of results with other labs.
- ☐ The benefits of EE are outweighed by the cost of its interference with results.
- ☐ The added variability of EE requires using more animals.
- ☐ Unreliable data from mice housed with EE may lead to wasting animals’ lives and resources.
- ☐ Cage bedding and ad lib water and food are sufficient to supply animals’ basic needs, while EE unnaturally extends survival
- ☐ Other:

6. Please select the main reason(s) leading your choice: *[Only answer this question if the following conditions are met: Answer was “Environmental Enrichment”. ' at question '4 [Q0004]'**]***

Please choose all that apply:

- ☐ EE does not confound results if provided to both experimental and control groups
- ☐ Treatment efficacy must be higher than therapeutic effect of EE.
- ☐ It is impossible to replicate the exact setting from lab to lab. If results are robust, they will be reproducible in slightly varying settings.
- ☐ The benefits of EE outweigh any potential variance in results
- ☐ If EE is confirmed to add variability (i.e. in a pilot), this can be balanced by increasing “n” per group and improving experimental design
- ☐ Unreliable data from standard housed mice may lead to wasting animals’ lives and resources.
- ☐ Animals in non-enriched cages show an unnaturally accelerated phenotype as a result of sensorial deprivation.
- ☐ Other:

[NOTE: QUESTIONS 7-13 NOT INCLUDED IN THIS STUDY**]**

14. Please read the following case-study:

Amyotrophic lateral sclerosis (ALS) is a neurodegenerative disease that causes progressive motor impairment, culminating in early death. Transgenic mouse models of ALS present a similar phenotype, showing progressive limb paralysis and a short life-span. A pre-clinical trial of a drug – delivered after disease onset – aims to test whether it can alleviate symptoms and delay disease progression.

Which of the following endpoints would you choose for this study? (Choose the option that better fits your view) *

Please choose only one of the following:

- ○ Spontaneous death. A measure of survival is needed, and this is the endpoint that better represents what happens in the clinical setting.
- ○ Euthanasia of moribund, unresponsive animals. Animals are provided palliative care (hydration and mash food) up to this point to prevent deaths from malnutrition and models the clinical setting more closely.
- ○ Euthanasia upon reaching a predefined clinical score. Parameters include motor impairment, body weight loss and difficulty to breathe. Palliative care is provided (hydration and mash food).
- ○ Euthanasia of animals no longer able to reach the food hopper, preventing animals from dying of starvation and dehydration. This endpoint can be used as a surrogate for death in survival studies.
- ○ Spontaneous death of animals to which palliative care (hydration and mash food) is provided. This prevents deaths from malnutrition, and models the clinical setting more closely.
- ○ Euthanasia of moribund, unresponsive animals. This allows a good approximation to real survival time, while averting spontaneous death. It also prevents cadaver decay from compromising biological samples.

15. Please read the following case-study:

A study on a rat model of a respiratory infection requires frequent body temperature checks, for an extended period of time. To avoid excessive handling, researchers have decided to implant a one centimetre-long telemetric device subcutaneously to measure body temperature without disturbing the animals. The procedure takes around 20 minutes per rat.

Should post-operative analgesia be given to these animals? *

Please choose only one of the following:

- ○ No. This is a small, fast procedure in which the implant is placed just under the skin.
- ○ Yes, as long as controls are sham-operated and given the same drugs for the same period of time.
- ○ Yes, we should always reduce any post-operative pain, even if it can interfere with our data.
- ○ No. Any post-operative pain is likely to be mild and analgesics may affect body temperature and skew the results.

16. How familiar are you with the most recent European directive on the protection of animals used in the life sciences, or its transposition to your national legislation? *

Please choose only one of the following:

- ○ I’m quite knowledgeable of the Directive and all of the changes brought about to research
- ○ I know the Directive fairly well, but I’m still not familiar with all the changes it has conveyed
- ○ I’m aware of some of the changes imposed by the Directive, but I do not know it in detail
- ○ I still do not know much about the 2010/63/EU directive

17 Regarding the regulation of animal use in the life sciences, please rate your degree of agreement with the following sentences: *
Please choose the appropriate response for each item:

	Strongly disagree	Disagree	Neither agree nor disagree	Agree	Strongly agree	Don’t Know
Legislation limits my freedom, as a researcher	○	○	○	○	○	○
Extensive regulation imposes a bureaucratic burden that slows down the research process	○	○	○	○	○	○
Extensive regulation may prevent the carrying out of relevant and necessary research	○	○	○	○	○	○
Strict regulations are needed to prevent malpractice	○	○	○	○	○	○
I trust researchers in my field of research act responsibly and according to high standards in animal welfare	○	○	○	○	○	○
I trust researchers in the country where I work act responsibly and according to high standards in animal welfare	○	○	○	○	○	○
Committees responsible for evaluating animal research projects should include ‘lay members’ (i.e. from the ‘general public’)	○	○	○	○	○	○
Representatives of animal protection groups should take part in the ethical evaluation of animal research projects	○	○	○	○	○	○

	Strongly disagree	Disagree	Neither agree nor disagree	Agree	Strongly agree	Don't Know
Scientists and institutions should be transparent regarding how and for what purpose animals are used in research	○	○	○	○	○	○
I would be willing to speak openly on how and why and I use animals to the public	○	○	○	○	○	○

18. Each researcher is, ultimately, the one responsible for how animals under his responsibility are treated. Scientists' attitudes to the principles of Replacement, Reduction and Refinement may however be influenced by a number of factors.

Please rate the level of importance which of the following factors has on how you use and treat animals.

(If you have not yet worked with laboratory animals, please select "not applicable"):
*
Please choose the appropriate response for each item:

	1 (Not important)	2	3	4	5 (Very important)	Don't Know	Not applicable
External pressure by animal protection groups	○	○	○	○	○	○	○
Editorial policies of scientific journals on animal welfare and ethics.	○	○	○	○	○	○	○
Feedback from editors and reviewers of scientific journals on animal welfare issues	○	○	○	○	○	○	○
Advice and supervision by animal care staff and veterinarians in the institution	○	○	○	○	○	○	○
Advice and supervision by Institutional Animal Welfare Bodies or Ethics Committees	○	○	○	○	○	○	○
Regulatory requirements (e.g. legislation)	○	○	○	○	○	○	○
Evaluation of research projects by funders	○	○	○	○	○	○	○
Guidelines on animal use from within the scientific community in your field of research	○	○	○	○	○	○	○
Training courses in Laboratory Animal Science	○	○	○	○	○	○	○

19. How would you rate what **what you have learned** (first column) and the **usefulness to your own work** (second column) of the following topics addressed in the course? *

Please choose the appropriate response for each item:

	I did not learn anything of relevance on this topic	I know somewhat more than I did, from what I learned in the course	I know substantially more than I did, from what I learned in the course	What I learned has had no impact on my current work	What I learned on this topic had some influence on my work with animals	What I learned profoundly influenced my work with animals
Replacement alternatives to animal experiments	O	O	O	O	O	O
Reduction of the number of animals used for each experiment	O	O	O	O	O	O
Refinement of procedures to minimize harm and improve wellbeing	O	O	O	O	O	O
Identification of signs of pain or distress	O	O	O	O	O	O
Design of animal experiments	O	O	O	O	O	O
Scientific validity and integrity of animal experiments	O	O	O	O	O	O
Handling techniques	O	O	O	O	O	O
Substance administration techniques	O	O	O	O	O	O
Sampling techniques	O	O	O	O	O	O
Anaesthesia and post-operative analgesia	O	O	O	O	O	O
Euthanasia and humane end-points	O	O	O	O	O	O

20. Please feel free to leave any comments about this questionnaire or your experience with training in laboratory animal science.

Please write your answer here:

Thank you for your collaboration!

Submit your survey.

Thank you for completing this survey.
